# Exploring Phytochemical Composition and In Vivo Anti-Inflammatory Potential of Grape Seed Oil from an Alternative Source after Traditional Fermentation Processes: Implications for Phytotherapy

**DOI:** 10.3390/plants12152795

**Published:** 2023-07-28

**Authors:** Yancho Zarev, Lyubomir Marinov, Denitsa Momekova, Iliana Ionkova

**Affiliations:** 1 Department of Pharmacognosy, Faculty of Pharmacy, Medical University of Sofia, 2 Dunav Str., 1000 Sofia, Bulgaria; ionkova@pharmfac.mu-sofia.bg; 2 Department of Pharmacology, Pharmacotherapy, and Toxicology, Faculty of Pharmacy, Medical University of Sofia, 2 Dunav Str., 1000 Sofia, Bulgaria; lmarinov@pharmfac.mu-sofia.bg; 3 Department of Pharmaceutical Technology and Biopharmaceutics, Faculty of Pharmacy, Medical University of Sofia, 1000 Sofia, Bulgaria; dmomekova@pharmfac.mu-sofia.bg

**Keywords:** grape seed oil (GSO), alternative source, in vivo, anti-inflammatory effects, GC-HR-EIMS analysis

## Abstract

This study aimed to analyze the composition of grape seed oil (GSO) derived from an alternative source after traditional fermentation processes and its potential anti-inflammatory effects using an in vivo model of carrageenan-induced inflammation in mice. Gas chromatography high-resolution electron ionization mass spectrometry (GC-HR-EIMS) analysis identified eight main components in the GSO extract, including myristic acid methyl ester, palmitoleic acid methyl ester, methyl isoheptadecanoate, cis-linoleic acid, oleic acid methyl ester, linoleic acid stereoisomer, linoleic acid ethyl ester, and methyl (6*E*, 9*E*, 12*E*, 15*E*)-docose-6,9,12,15-tetraenoate. No significant differences were observed in the main fatty acids between commercially available grape seed oil and GSO extract obtained from fermented grape seeds. In the carrageenan-induced inflammation model, treatment with GSO resulted in a significant reduction in paw edema at 180 min, as in the reduction observed with diclofenac treatment. Combined treatment with GSO and diclofenac showed enhanced anti-inflammatory effects. Additionally, GSO exhibited antioxidative effects by decreasing the levels of glutathione (GSH) and malondialdehyde (MDA) in the serum. Chronic treatment with GSO for ten days did not provide a protective effect on inflammation. These findings suggest that GSO could be used as an alternative raw material and could possess anti-inflammatory and antioxidative properties. Further studies are needed to explore its potential therapeutic applications.

## 1. Introduction

*Vitis vinifera* is a perennial climbing plant native to Southern Europe and Western Asia, and is currently cultivated worldwide. It belongs to the family Vitaceae. All members of the genus *Vitis* are climbing vines or woody plants. The genus *Vitis* is primarily found in temperate and subtropical climatic zones of the Northern Hemisphere [1]. Grape seed oil (GSO) is one of the richest natural sources of tocopherols, which are among the most powerful fat-soluble antioxidants. The oil has been shown to support the treatment of cardiovascular diseases and malignant tumors [2]. Regarding phytosterols in GSO, the highest concentration is *β*-sitosterol, at up to 65%, followed by stigmasterol at around 10%. The interest in phytosterols originates from their antioxidant activity and their role in cholesterol metabolism. Specifically, *β*-sitosterol in combination with polyphenols derived from winemaking has demonstrated cardioprotective activity in vitro by preventing the release of inflammatory mediators and atherogenic molecules [3]. GSO from *Vitis vinifera* is commonly obtained through the cold pressing method to retain the antioxidant components; however, GSO has been extracted using organic solvents such as hexane as well (Soxhlet extraction). Supercritical fluid extraction (SFE) utilizing carbon dioxide (CO_2_) is an environmentally friendly and economically efficient alternative that yields a superior product in comparison to mechanical pressing methods. Although the yield is lower than that of hexane extraction or cold pressing, this method can be enhanced through pre-enzymatic treatment of the seeds [4]. Other methods of oil extraction include pressurized liquid extraction (PLE), microwave-assisted extraction (MAE), and ultrasound-assisted extraction (UAE) [5,6,7].

The fruits of the grapevine are rich in polyphenols (anthocyanins, flavonols, stilbenes), phenolic acids, proteins, lipids, and vitamin C. Grape seed extract contains the compounds procyanidins, gallic acid, epicatechin, catechin, and quercetin. Grape seed extract is rich in phenolic compounds as well, including caffeic acid, coumaric acid, ferulic acid, and tartaric acid, along with rutin, quercetin-3-*O*-*β*-D-glucoside, quercitrin, myricetin, catechin, and epicatechin. Grape seeds contain 38.2% fiber, 15.80% total lipids, 10.70% protein, 2.58% ash, 10.40% moisture, and 22.37% carbohydrates. The extract from grape roots contains the stilbenoid compounds resveratrol, vitisin A and B, and miyabenol C, as indicated by Esatbeyoglu et al., 2016 [8,9]. Resveratrol is considered the most important of the stilbenoids found in plants. Resveratrol exhibits antioxidant, anticancer, anti-inflammatory, antidiabetic, and overall antimicrobial effects. Other stilbenoid compounds found in the roots include trans-piceid, cis-piceid, vitisinol B, ampelopsin C, and ampelopsin E [1].

Both the hydrophilic and lipophilic components present in GSO play a significant role in ameliorating inflammatory processes associated with various chronic diseases. Phenolic compounds have been shown to exert their effects through the modulation of gene expression, specifically influencing inflammatory cellular pathways involved in the release of arachidonic acid, cytokine production, and nitric oxide [10]. The intricate interplay between inflammation and insulin resistance further underscores the impact of inflammatory processes on metabolic disorders. Notably, several studies have demonstrated the beneficial effects of GSO, attributing them to the presence of phenolic compounds and tocotrienols. *β*-sitosterol, a prominent phytosterol found in GSO, exhibits protective properties by effectively inhibiting the release of inflammatory mediators during pro-inflammatory conditions. It accomplishes this by stimulating macrophages through the utilization of oxidized low-density lipoproteins (LDL) and regulating eicosanoid synthesis [11]. The multifaceted mechanisms through which *β*-sitosterol exerts its anti-inflammatory effects highlight its potential as a therapeutic agent. Furthermore, recent findings have shed light on the anti-inflammatory properties of linoleic acid, a key component of GSO, particularly in animal cells. Linoleic acid has demonstrated its natural capacity to mitigate inflammation, offering promising prospects for combating inflammatory-related pathologies [12].

Markoski et al., 2016 claimed that wine retains various organic compounds derived from grapes even after undergoing fermentation, including polysaccharides, acids, and phenolic compounds comprising both flavonoids and non-flavonoids [13]. Considering the valuable qualities of GSO, the present study aims to find an alternative source for obtaining oil, specifically, by utilizing grape seeds obtained as a waste product after traditional fermentation processes. Following the extraction of the oil and its fractionation, a comparative analysis using combined techniques (GC-HR-EIMS) is used to assess the quality of the resulting oil compared to oil available on the market for culinary purposes. The results from studies on extracts rich in proanthocyanidins and fatty acids are frequently confusing, and often present mixed data in the same studies [14]. Therefore, further research is necessary to conduct in-depth analysis of the pharmacological activity of GSO. The findings derived from the comparative quality analysis presented in this paper can enhance our understanding of the phytochemical profile of grape seed oil following different fermentation processes, thereby shedding light on their viability as an alternative source for oil extraction. Furthermore, the outcomes of pharmacological investigations are anticipated to unravel the advantageous characteristics of grapeseed oil as a natural product, unveiling its potential benefits.

## 2. Results

### 2.1. Fatty Acid Profile

As a result of the GC-HR-EIMS analysis of the obtained GSO and oil available on the market for culinary purposes, eight main components were identified: myristic acid methyl ester (C_14:0_), palmitoleic acid methyl ester (C_16:1n-7_), methyl isoheptadecanoate (C_16:0_), cis-linoleic acid (C_18:2n-6,9_), oleic acid methyl ester (C_18:1n-9_), linoleic acid stereoisomer, linoleic acid ethyl ester (C_18:2-n-6,9_), methyl (6*E*, 9*E*, 12*E*, 15*E*)-docose-6,9,12,15-tetraenoate (C_22:4n-7,10,13,16_), and cis-methyl 11-eicosenoate (C_20: 1n-9_) (Table 1). No significant differences were observed for the main fatty acids in the commercially available GSO (B) and the GSO (A) obtained from grape seeds after fermentation processes, with the exception of 6,9,12,15-docosatetraenoic and linoleic acids. The identified compounds were identical for both oils, indicating that fermented grape seeds could be used as an alternative raw material.

### 2.2. Anti-Inflammatory Activity Study

**Figure 1:**
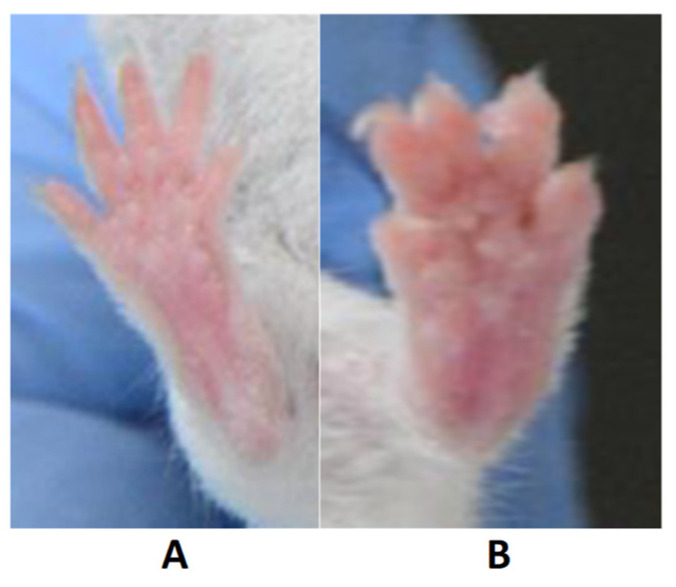
Representative visualization of paw edema: (**A**) Group I, control group and (**B**) Group II, control carrageenan group.

**Figure 2:**
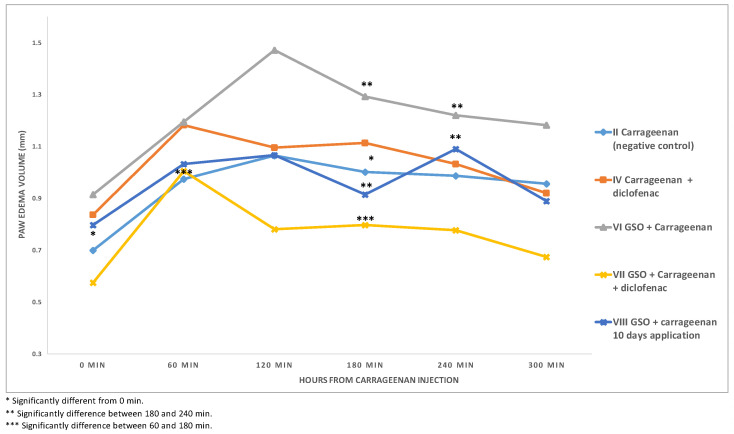
Paw edema size during anti-inflammatory activity study after carrageenan-induced inflation.

Figure 1 and Figure 2 shows that mice in each group injected with 1% carrageenan showed signs of inflammation (paw oedema) at 60, 120, 180, 240, and 300 min after injection, respectively (Figure 2; Table 2). Each group contained six animals. The figure does not show the groups where no inflammation was induced, as in those groups there was no change in the paw volume within the time frame of 300 min. At 180 min, in the negative control group (II) the inflammation was significantly different from baseline (0 min or starting point). When the mice were treated with GSO (group VI), a significant reduction in the inflammation of the paw oedema was observed at exactly time 180 min, which is considered the most important point for inflammation. It should be noted that the paw volume of group VI at the start of the experiment was the highest; this is related to the fact that this group had bigger paws. The same magnitude of reduction in paw oedema was observed at 180 min when combined treatment with GSO and diclofenac was applied. Chronic treatment with GSO for ten days showed no protective effect against inflammation, as the most significant effect of reducing paw oedema was observed at the same time point (180 min).

### 2.3. Turbidity Test Results

**Figure 3:**
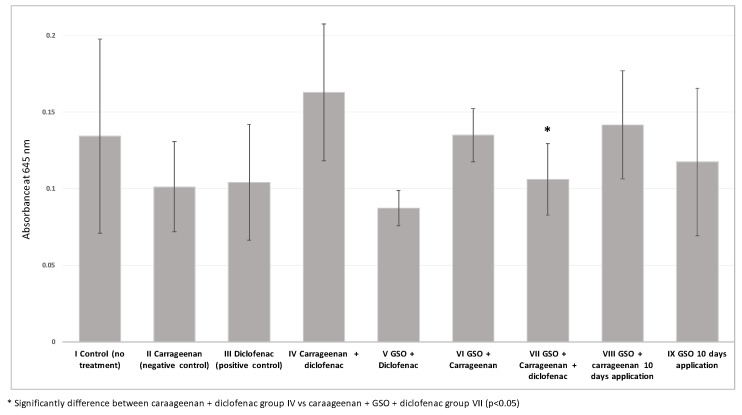
Results of turbidity test for all groups.

As shown in Figure 3, the improvement of inflammation based on significant decrease in serum turbidity was recorded in group (VII) compared with group (IV). Thus, combined treatment with diclofenac and GSO could be considered to have more beneficial effects related to carrageenan-induced inflammation (Figure 3).

### 2.4. Changes in Oxidative Stress Biomarkers 

**Figure 4:**
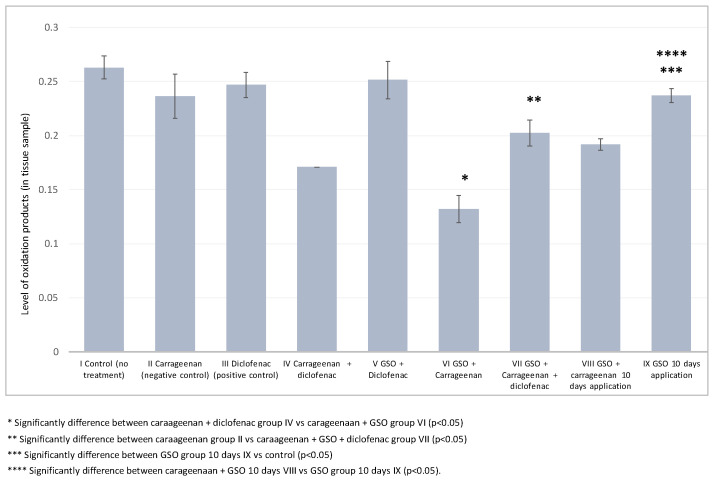
Paw tissue oxidative damage biomarker (GSH) for mice from all groups.

**Figure 5:**
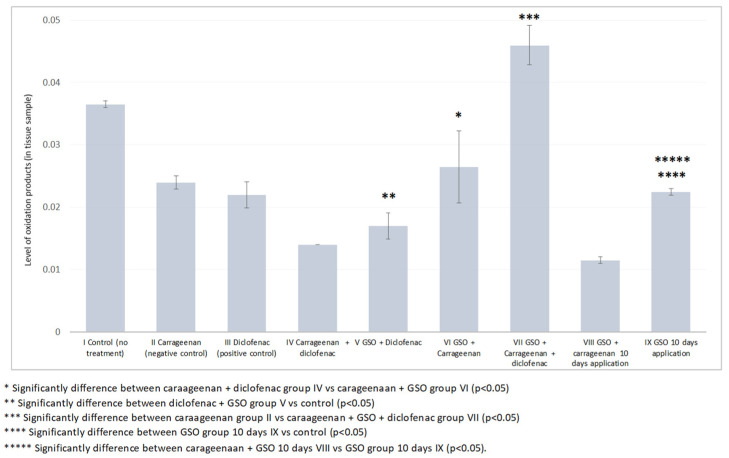
Paw tissue oxidative damage biomarker (MDA) for mice from all groups.

The oxidative stress biomarker GSH showed significantly decreased levels in group (VI) compared to group (IV), which is considered as being due to the antioxidative effect of GSO when inflammation was induced with 1% carrageenan. This strong antioxidative effect of GSO was observed when it was applied together with diclofenac in group (VII) vs. the negative control, group (II), with respect to GSH. In addition, the most powerful antioxidative effect of GSO was observed after chronic exposure for ten days, in group (IX). Significant degrees of the GSH and MDA biomarkers were noticed in the comparison vs. the control group. In confirmation of this statement is the observed statistically significant decrease in the levels of GSH and MDA during chronic administration of GSO in group (IX) and induced inflammation with carrageenan in group (VIII) (Figure 4 and Figure 5).

### 2.5. Clinico-Biochemical Results

A comparison between the control group (I) vs. the carrageenan group (II) showed a significantly increased level of cholesterol. Serum levels of T-BIL, ALAT, CHOL, and TRIG in the diclofenac treated mice of group (III) differed significantly from the control group (I). On the other hand, compared to the negative control carrageenan group (II), the carrageenan + diclofenac group (IV) showed marked elevation in serum T-BIL and D-BIL levels, an observation which was found for the carrageenan + GSO group in respect to T-BIL, L-BIL, ASAT, ALAT, and TRIG (Table 3). 

## 3. Material and Methods

### 3.1. General Experimental Procedures

Hexane, chloroform, and methanol of HPLC grade were purchased from Fisher Chemicals. KOH-MeOH solution (0.5 M) was obtained from Supelco (Titripur^®^, Darmstadt, Germany). *λ*-Carrageenan (Sigma-Aldrich, St. Louis, MN, USA) 1% solution was prepared by dissolving carrageenan in saline (0.9% NaCl). The solution (0.1 mL) was injected subplantarly into the hind paw of experimental animals in groups II, IV, VI, VII, and VIII. Diclofenac sodium (Fluka) was suspended in 0.5% aqueous solution of sodium carboxymethyl cellulose and administered by oral gavage in a concentration of 0.2 mL (100 mg/kg).

GC-MS analysis of the (fatty acids methyl esters mixture) FAME was performed using a Hewlett Packard 5890 gas chromatograph (Hewlett Packard, Palo Alto, CA, USA) connected to a VG Autospec (VG Analytical Ltd., Manchester, UK) high-resolution magnetic instrument (double focusing) operating in total ion current mode (TIC) at 70 eV with a resolving power of 10,000. GC-HR-EIMS analysis of FAME was performed using an Exactive™ Orbitrap™ GC-MS (Thermo Fisher Scientific, Bremen, Germany) system operating at 70 eV, an ion source temperature of 230 °C, a transfer capillary temperature of 260 °C, and split injection (1 μL, 20:1 ratio) at an injector temperature of 250 °C. Helium was used as the carrier gas (flow rate: 1 mL/min). The parameter settings of the gas chromatograph were as follows: a capillary column with 5% phenyl residues/95% methylpolysiloxane (TraceGOLD TG -5SilMS GC Column 30 m × 0.25 mm × 0.25 µm, Thermo), injection volume 2 µL, and column split ratio 1:100; the oven temperature program was initially set at 110 °C for 5 min, increased to 205 °C (rate: 4 °C/min), increased to 215 °C (rate: 1 °C/min), and held at 250 °C for 5 min. EI ionization mode and a full MS-selected ion monitoring (SIM) scan were used (resolution 600, AGC target 1e6, maximum injection time (IT) 200 ms, and scan range from *m*/*z* 50 to *m*/*z* 450).

### 3.2. GSO Extraction

The grape seeds used were of the Muscat *Ottonel* variety, obtained after a fermentation process in the traditional production of spirits at home. The dried seeds (175.00 g) were extracted with 6 L of hexane. The extraction process coincided with the crushing of the seeds, for which the IKA T25 digital ULTRA TURRAX was used. The obtained extract was filtered through a filter paper (0.22 μm) and concentrated. The resulting mixture was dissolved in hexane and applied to a flash chromatography column (50 cm × 3.5 cm) against silica gel with a particle size 40–63 µm and a pore size of 60 Å (Merck, Darmstadt, Germany). Elution was performed with hexane (1 L) only, resulting in a purified oil fraction (GSO). The GSO was suspended in Tween 80 and administered orally by gavage to the experimental animals at a concentration of 5 mg/kg body weight (bw). The same extract was used for GC/MS analysis. For conducting the comparative analysis, oil available in the markets was used (the brand name of the oil used is not mentioned here to avoid any conflict of interest).

### 3.3. Methylation Method for FAME (Fatty Acids Methyl Esters) Synthesis

GSO (40 µL) was transferred into 10 mL glass tubes with Teflon-coated lids. Before determining the fatty acid composition by gas chromatography, it is necessary to hydrolyze the lipids and subsequently methylate the released fatty acids to methyl esters in order to increase their volatility. Therefore, 3 mL of KOH-MeOH solution (0.5 M) was added to the vials and the mixture was heated at 60 °C for 15 min [15]. The hydrolysate was cooled and 2 mL of 4N HCl in H_2_O and 3 mL of hexane/chloroform (1:1) were added. The organic layer was washed with 3 mL 0.24 M KOH and dried over anhydrous Na_2_SO_4_, then 2 µL extracted solution was injected into GC-HR-EIMS. The same methylation procedure was performed with the grape seed oil available from the market.

### 3.4. In Vivo Experimental Protocol

Adult male mice (54 pieces, weight 20–30 g) were purchased from the vivarium in Slivnitsa, Bulgaria. The animals were acclimatized for 10 days in transparent plexiglas cages (20/10/15 cm) in the vivarium of the Faculty of Pharmacy at the Medical University of Sofia, Bulgaria. A 12 h light–dark cycle controlled by an automatic timer was used. The animals had free access to food and water. The air temperature was maintained at 22 ± 3 °C and humidity at 60% ± 4%. All procedures performed were approved by the Bulgarian Food Safety Authority (BFSA) No. 342, and the principles of the European Convention for the Protection of Vertebrate Animals used for Experimental and other Scientific Purposes (ETS 123) (Council of Europe, 1991) were strictly followed throughout the experiment.

After conditioning, the animals were randomly divided into nine groups of six mice each. Group I: control group without any treatment; Group II: control carrageenan group injected with 0.1 mL of 1% carrageenan solution subplantarly into the left hind paw for 1 day; Group III: received 0.2 mL (100 mg/kg) of diclofenac by oral gavage for 1 day; Group IV: injected with 0.1 mL of a 1% carrageenan solution and 0.2 mL (100 mg/kg) diclofenac administered by oral gavage for 1 day; group V: received 0.2 mL diclofenac (100 mg/kg) + 5 mg/kg bw GSO by oral gavage for 1 day; group VI: injected with 0.1 mL of 1% carrageenan solution and received 5 mg/kg bw GSO via oral gavage for 1 day; group VII: injected with 0.1 mL of 1% carrageenan solution and received 0.2 mL of diclofenac (100 mg/kg bw) + 5 mg/kg bw GSO via oral gavage for 1 day; group VIII: injected with 0.1 mL of 1% carrageenan solution after receiving 5 mg/kg bw GSO via oral gavage for 10 days; group IX: received 5 mg/kg bw GSO by oral gavage for 10 days. The paw volume was measured for each group with induced inflation after 1, 2, 3, 4, and 5 h post-carrageenan exposure.

### 3.5. Determination of Serum Biochemical Markers

Within 30 min of collection, blood samples obtained after decapitation of the animals were centrifuged (Eppendorf MiniPlus) at 1500 rpm for 10 min at a temperature of 4 °C. Immediately after centrifugation, the resulting plasmas were separated and stored at −20 °C until analysis. The biochemical analysis tracks eight parameters: total bilirubin (T-BIL), direct bilirubin (D-BIL), aspartate aminotransferase (ASAT), serum alanine aminotransferase (ALAT), alkaline phosphatase (ALP), gamma-glutamyl transferase (GGT), cholesterol (CHOL), and triglycerides (TRIG). Serum biochemical markers (six samples from each group) were analyzed, and the data are presented as mean ± S.E.M.

### 3.6. Serum Turbidity Test

The serum (0.1 mL) was mixed with 2.9 mL of 0.067 mol/L Sorensen buffer (a mixture of 2 mL of 0.067 mol/L potassium hydrogen phosphate and 98 mL of 0.067 mol/L disodium hydrogen phosphate at pH = 5.2). The mixture was stabilized at room temperature for 15 min and then incubated in a water bath at 69 °C for 30 min. The tubes were cooled in an ice bath and the absorbance was measured at 645 nm [16].

### 3.7. Determination of Oxidative Stress Biomarkers

The paw tissue (0.50 g) was crushed in a mortar with liquid nitrogen and homogenized with 0.1 M phosphate buffer and EDTA, pH = 7.4 (1:10). The method was described and modified by Polizio and Pena, 2005 [17]. To the homogenate was added 1 mL of 25% trichloroacetic acid (TCA) and 1 mL of 0.67% thiobarbituric acid. After heating, collaging and centrifugation, the absorbance of the supernatant was measured at 535 nm. The MDA concentration was calculated using a molar extinction coefficient of 1.56 × 105 M^−1^ cm^−1^ and expressed in nmol/g wet tissue. Similarly, for GSH the paw tissue was homogenized with 5% TCA and centrifuged. The supernatant was then mixed with phosphate buffer and 0.02 mL of 2,2-dinitro-5,5-dithiodibenzoic acid. Absorbance was measured at 412 nm and the results expressed in nmol/g wet tissue [18].

### 3.8. Investigation of the Degree of Inflammation by Measuring Paw Edema Size with Plethysmometer

A plethysmometer consists of two cylinders connected through the principle of docked vessels, one of which is equipped with a pair of electrodes. We used a model 7140 plethysmometer (Ugo Basile, Gemonio, Italy). During measurement, the vessels were filled with liquid electrolyte as a wetting agent in concentrations of 2–3 mL/L, as included in the standard package. When immersing the paw of the test animal up to the hairline, the level of the liquid changes, resulting in a change in conductivity between the two electrodes. The recorded changes in water displacement are shown on an electronic display in milliliters, and were recorded for analysis.

### 3.9. Data Analysis

Data acquisition and peak processing after GC/MS analysis was performed using an Xcalibur 4.2.28.14 (Thermo Scientific, Bremen, Germany). The major chemical components were identified by comparing their exact mass spectra with those of the NIST mass spectral library. Statistical analysis was performed using SPSS. The equality of variance was checked with Levene’s test, and the independent sample *t*-test was performed for equality of means. The level of statistical significance was set at *p* < 0.05. The results are presented as means ± ST.DEV.

## 4. Conclusions

Based on the results of paw oedema-induced inflammation, experimental mice administered 1% carrageenan showed a marked inflammatory response characterized by the development of paw oedema at various time intervals after injection. Paw oedema in the negative control group (group II) increased after 180 min. In contrast, treatment with the GSO extract (group VI) resulted in a remarkable decrease in paw oedema at exactly the 180 min time point, which is of particular importance in terms of progression of inflammation, as at this timepoint the negative control group (II) showed a level of inflammation significantly different from baseline (0 min or starting point). This indicates the potential anti-inflammatory effect of GSO in alleviating paw oedema. In addition, combined treatment with GSO and diclofenac showed a significant reduction in paw oedema after 180 min, further supporting the anti-inflammatory properties of GSO. However, it is worth noting that chronic administration of GSO over a period of ten days did not show a sustained protective effect against inflammation. Despite the prolonged treatment, the most marked reduction in paw oedema was observed at the acute time point of 180 min. These results suggest that although GSO has an acute anti-inflammatory effect, it has limited efficacy in providing sustained protection against inflammation. Further comprehensive studies are needed to decipher the underlying mechanisms responsible for the anti-inflammatory effect of GSO extract and to explore its potential therapeutic applications in inflammatory diseases.

In addition to the above results, a serum turbidity assessment provided further evidence of improvement in inflammation. In particular, the group receiving the combined diclofenac and GSO treatment (group VII) showed a significant decrease in serum turbidity compared to group IV. This observation suggests that combined treatment with diclofenac and GSO can better alleviate the inflammation triggered by carrageenan. By combining the anti-inflammatory properties of diclofenac with the potential anti-inflammatory effects of GSO, a synergistic effect can be achieved, leading to a more marked improvement in inflammation-related outcomes. These results highlight the potential of combining GSO with conventional anti-inflammatory agents to optimize therapeutic efficacy and potentially reduce the dosage or duration of drug administration. However, further studies are needed to elucidate the exact mechanisms underlying these synergistic effects and to explore the long-term impact of such combined treatments.

The observed significant decrease in GSH levels in group VI, in which GSO was administered during inflammation induced by carrageenan, suggests that GSO exerts an antioxidant effect. The decrease in GSH levels indicates increased scavenging of reactive oxygen species (ROS) and protection against oxidative stress in the inflamed paws. This finding supports the assumption that GSO has strong antioxidant properties and can effectively mitigate the harmful effects of oxidative stress during inflammation. Furthermore, combined treatment with GSO and diclofenac (group VII) showed a strong antioxidant effect, as evidenced by the significant decrease in GSH levels compared to the negative control (group II). This suggests that GSO synergistically enhances the antioxidant effect of diclofenac, a well-known anti-inflammatory drug. Such combined treatment could provide a more comprehensive approach to combating inflammation-induced oxidative stress, offering potential benefits in the treatment of inflammatory conditions. Furthermore, the strongest antioxidant effect of GSO was observed after chronic exposure for ten days (group IX). This suggests that prolonged administration of GSO increases its antioxidant capacity, leading to a more pronounced reduction in GSH levels. The significant degrees of GSH and MDA biomarkers observed in comparison to the control group (group II) further support the antioxidant effect of GSO during chronic administration and inflammation induced by carrageenan (group VIII). These results suggest that the antioxidant potential of GSO is maintained over time and underpins its role in combating oxidative stress associated with inflammation.

Overall, our results show that GSO has a considerable antioxidant effect by lowering GSH levels, indicating enhanced ROS scavenging and protection against oxidative stress in the inflamed paws of the mice tested in this study. This antioxidant effect is evident when GSO is administered alone both and in combination with diclofenac. Furthermore, chronic administration of GSO further enhances its antioxidant properties. These results highlight the therapeutic potential of GSO as a natural antioxidant in the management of inflammation-induced oxidative stress, and can provide insights into its mechanisms of action in the context of inflammatory diseases.

The comparison between the control group (Group I) and the mice in the carrageenan group (Group II) revealed a significant increase in the level of cholesterol. This suggests that carrageenan-induced inflammation may contribute to alterations in lipid metabolism, leading to elevated cholesterol levels. In the Group III mice treated with diclofenac there were significant differences in the serum levels of T-BIL, ALAT, CHOL, and TRIG compared to the control group (Group I). These findings indicate that diclofenac administration impacts these biochemical markers, potentially influencing liver function and lipid metabolism. The marked elevation in serum levels of T-BIL (total bilirubin) and D-BIL (direct bilirubin) in the carrageenan + diclofenac group (Group IV) compared to the negative control carrageenan group (Group II) suggests that combined treatment with carrageenan and diclofenac may influence bilirubin metabolism and liver function. Similarly, in the carrageenan + GSO group significant differences were observed in T-BIL, L-BIL, ASAT, ALAT, and TRIG levels compared to the negative control carrageenan group. These results indicate that GSO administration in the presence of carrageenan-induced inflammation affects these biochemical markers, potentially influencing liver function and lipid metabolism. Overall, our findings highlight the impact of carrageenan-induced inflammation, diclofenac treatment, and GSO administration on various biochemical markers related to liver function and lipid metabolism. The observed alterations in these markers suggest potential effects on hepatic health and lipid homeostasis which may have implications for understanding the mechanisms underlying inflammation-associated metabolic changes. Further investigations are warranted to elucidate the specific mechanisms involved along with their clinical significance.

## Figures and Tables

**Table 1:** Comparative GC/MS analysis of GSO obtained from grape seed (A) and oil commercially available for culinary purposes (B).

Fatty Acid Profile	A	B
Myristic acid (C_14:0_)	+	+
Palmitoleic acid (C_16:0_)	+	+
Isoheptadecanoic acid (C_16:1n-7_)	+	+
Cis-linoleic acid (C_18:2n-6,9_)	+	+
Oleic acid (C_18:1n-6,9_)	+	+
Linoleic acid (C_18:2n-9_)	+	-
Methyl (6*E*, 9*E*, 12*E*, 15*E*)-docose-6,9,12,15-tetraenoate (C_22:4n-7,10,13,16_)	+	-
Cis-methyl 11-eicosenoate (C_20:1n-9_)	+	+

**Table 2:** Paw edema size during anti-inflammatory activity study after carrageenan-induced inflation groups, presented as means ± ST.DEV.

	Groups	0 min	60 min	120 min	180 min	240 min	300 min
II	Carrageenan (negative control)	0.7000 ± 0.1217	0.9742 ± 0.2409	1.0650 ± 0.3445	1.0025 ± 0.2286 *	0.9867 ± 0.2120	0.9575 ± 0.1904
IV	Carrageenan + diclofenac	0.8375 ± 0.1382	1.1833 ± 0.3785	1.0950 ± 0.2669	1.1142 ± 0.3660	1.0317 ± 0.3098	0.9200 ± 0.2290
VI	Oil + Carrageenan	0.9155 ± 0.1782	1.1955 ± 0.2116	1.4718 ± 0.2596	1.2927 ± 0.3445 **	1.2209 ± 0.2606 **	1.1836 ± 0.2723
VII	Oil + Carrageenan + diclofenac	0.5750 ± 0.2397	1.0050 ± 0.2266 ***	0.7817 ± 0.1710	0.7975 ± 0.2152 ***	0.7783 ± 0.2630	0.6742 ± 0.2979
VIII	Oil + carrageenan 10 days	0.7980 ± 0.1718	1.0330 ± 0.3206	1.0670 ± 0.2726	0.9150 ± 0.2759 **	1.0900 ± 0.2347 **	0.8890 ± 0.3999

* Significantly different from 0 min. ** Significantly difference between 180 and 240 min. *** Significantly difference between 60 and 180 min.

**Table 3:** Biochemical marker results for all groups.

	Groups	T-BIL (μmol/L)	D-BIL (μmol/L)	ASAT (U/L)	ALAT (U/L)	ALP (U/L)	GGT (U/L)	CHOL (mmol/L)	TRIG (mmol/L)
I	Control (no treatment)	12.52 ± 1.54	7.10 ± 1.75	348.68 ± 70.5	99.96 ± 13.83	118.40 ± 55.85	-	2.37 ± 0.20	1.35 ± 0.40
II	Carrageenan (negative control)	11.17 ± 2.36	5.00 ± 1.07	336.2 ± 75.85	96.42 ± 11.18	89.67 ± 13.47	-	2.89 ± 0.39 *	0.93 ± 0.14
III	Diclofenac (positive control)	8.58 ± 1.94 **	4.14 ± 0.99	296.88 ± 42.61	66.66 ± 7.25 **	75.00 ± 8.80	0.82 ± 1.86	2.13 ± 0.06 **	0.89 ± 0.13 **
IV	Carrageenan + diclofenac	17.35 ± 1.49 ***	9.05 ± 0.64 ***	602.35 ± 284.89	165.4 ± 50.63	79.00 ± 9.90	-	2.61 ± 0.55	1.15 ± 0.26
V	Oil + Diclofenac	6.00 ± 0.75	2.30 ± 0.41	131.67 ± 9.91	159.00 ± 8.56	61.67 ± 15.20	4.4 ± 2.61	2.65 ± 0.67	1.60 ± 0.47
VI	Oil + Carrageenan	15.43 ± 2.14 ****	6.68 ± 1.24 ****	574.08 ± 223.60 ****	198.43 ± 43.70 ****	103.50 ± 43.96	-	2.67 ± 0.48	1.16 ± 0.14 ****
VII	Oil + Carrageenan + diclofenac	19.18 ± 3.73	9.17 ± 2.86	370.65 ± 168.32	161.40 ± 65.39	93.67 ± 34.55	-	2.21 ± 0.57	1.37 ± 0.32
VIII	Oil + carrageenan 10 days	15.14 ± 3.11	7.88 ± 1.00	471.83 ± 38.81	113.10 ± 24.48	144.00 ± 44.89	-	2.48 ± 0.42	1.34 ± 0.10
IX	Oil 10 days application	16.96 ± 2.28	8.64 ± 14.07	457.52 ± 80.29	121.62 ± 21.62	148.40 ± 113.44	-	2.89 ± 0.61	1.32 ± 0.49

** Significantly different between diclofenac group III vs. control (*p* < 0.05). *** Significantly different between carrageenan group II and carrageenaan + diclofenac group IV (*p* < 0.05). **** Significantly different between carrageenan group II vs. carrageenan + GSO group VI.

## Data Availability

Data are available by request from the corresponding author (yzarev@pharmfac.mu-sofia.bg).
